# (Dicyanamido)[tris­(2-pyridylmeth­yl)amine]zinc(II) perchlorate

**DOI:** 10.1107/S1600536808004741

**Published:** 2008-02-27

**Authors:** Hong Li, Hong Yan Zhao, Shi Guo Zhang

**Affiliations:** a Department of Chemistry and Chemical Engineering, Institute of Materials Chemistry, Binzhou University, Binzhou 256603, People’s Republic of China; b Department of Chemistry, Shandong Normal University, Jinan 250014, People’s Republic of China

## Abstract

In the title complex, [Zn(C_2_N_3_)(C_18_H_18_N_4_)]ClO_4_, the Zn^II^ ion has a slightly distorted trigonal–bipyramidal ZnN_5_ coordination geometry. The crystal structure is stabilized by weak inter­molecular C—H⋯O and C—H⋯N hydrogen bonds. In addition, there are relatively close contacts between the O atoms of the perchlorate anion and symmetry-related pyridine rings [O⋯*Cg* = 3.179 (3) and 3.236 (3) Å, where *Cg* is the centroid of a pyridine ring], and between the terminal N atom of the dicyanamide ligand and pyridine rings [N⋯*Cg* = 3.381 (4)–3.761 (3) Å]. The central N atom of the dicyanamide ligand is disordered over two sites in an approximately 0.6:0.4 ratio.

## Related literature

For related literature, see: Makowska-Grzyska *et al.* (2003 ▶); Sun *et al.* (2003 ▶); Martin *et al.* (2001 ▶).
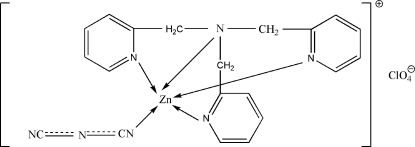


## Experimental

### 

#### Crystal data

[Zn(C_2_N_3_)(C_18_H_18_N_4_)]ClO_4_
*M* *_r_* = 521.23
Monoclinic, 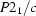

*a* = 13.931 (2) Å
*b* = 10.8578 (18) Å
*c* = 14.653 (2) Å
β = 91.590 (3)°
*V* = 2215.5 (6) Å^3^
*Z* = 4
Mo *K*α radiation
μ = 1.27 mm^−1^
*T* = 298 (2) K
0.30 × 0.20 × 0.18 mm

#### Data collection

Bruker SMART APEX CCD diffractometer
Absorption correction: multi-scan (*SADABS*; Sheldrick, 1996 ▶) *T*_min_ = 0.702, *T*_max_ = 0.803
12346 measured reflections
4800 independent reflections
3352 reflections with *I* > 2σ(*I*)
*R*_int_ = 0.035

#### Refinement

*R*[*F* ^2^ > 2σ(*F* ^2^)] = 0.043
*wR*(*F* ^2^) = 0.106
*S* = 0.99
4800 reflections
308 parameters
1 restraint
H-atom parameters constrained
Δρ_max_ = 0.36 e Å^−3^
Δρ_min_ = −0.25 e Å^−3^

### 

Data collection: *SMART* (Bruker, 1997 ▶); cell refinement: *SAINT* (Bruker, 1997 ▶); data reduction: *SAINT*; program(s) used to solve structure: *SHELXTL* (Sheldrick, 2008 ▶); program(s) used to refine structure: *SHELXTL*; molecular graphics: *SHELXTL*; software used to prepare material for publication: *SHELXTL*.

## Supplementary Material

Crystal structure: contains datablocks I, global. DOI: 10.1107/S1600536808004741/lh2594sup1.cif

Structure factors: contains datablocks I. DOI: 10.1107/S1600536808004741/lh2594Isup2.hkl

Additional supplementary materials:  crystallographic information; 3D view; checkCIF report

## Figures and Tables

**Table:** 

N1—Zn1	2.053 (2)
N2—Zn1	2.048 (2)
N3—Zn1	2.059 (2)
N4—Zn1	2.215 (2)
N5—Zn1	2.021 (3)

**Table:** 

N5—Zn1—N2	101.94 (10)
N5—Zn1—N1	100.96 (11)
N2—Zn1—N1	118.19 (9)
N5—Zn1—N3	100.98 (10)
N2—Zn1—N3	117.58 (9)
N1—Zn1—N3	112.99 (9)
N5—Zn1—N4	179.41 (10)
N2—Zn1—N4	78.60 (9)
N1—Zn1—N4	78.95 (9)
N3—Zn1—N4	78.55 (9)

**Table 2:** Hydrogen-bond geometry (Å, °)

*D*—H⋯*A*	*D*—H	H⋯*A*	*D*⋯*A*	*D*—H⋯*A*
C3—H3⋯O4^i^	0.93	2.53	3.272 (4)	138
C7—H7*B*⋯O2^ii^	0.97	2.59	3.455 (4)	148
C10—H10⋯O1^iii^	0.93	2.58	3.511 (5)	179
C13—H13*A*⋯N6^iv^	0.97	2.61	3.457 (5)	146
C16—H16⋯N7′^v^	0.93	2.48	3.412 (18)	178

Symmetry codes: (i) 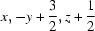; (ii) 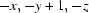; (iii) 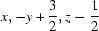; (iv) 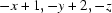; (v) 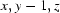.

**Fig. 1.**
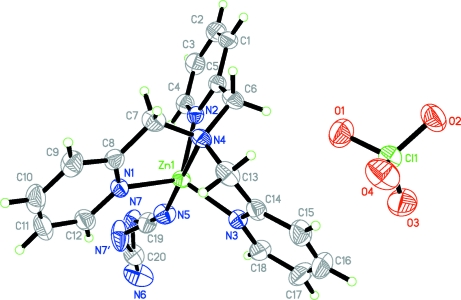
View of complex (I), showing the the atom numbering scheme with thermal ellipsoids drawn at the 30% probability level.

**Fig. 2.**
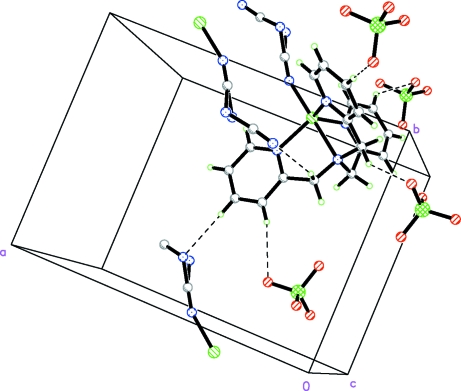
Part of the crystal structure showing weak hydrogen bonds as dashed lines.

## supplementary crystallographic information

### Comment

Tris[(2-pyridyl)methylene)amine is a very common terminal ligand and many complexes containing this ligand have been reported, including some Zn(II) complexes with perchlorate anions (*e.g.* Makowska-Grzyska *et al.*, 2003) such as in the title compound. The dicyanamide anion can play a role as bridging ligand to form multi-nuclear complexes (*e.g.* Sun *et al.*, 2003; Martin *et al.*, 2001). As part of our work we wanted to prepare a coordination polymer with dicyanamide group acting as a bridging ligand and tris[(2-pyridyl)methylene)amine as terminal ligand, but instead we obtained the title mononuclear five-coordinated Zn(II) complex (I) and its crystal structure is reported herein. At this time there appears to be no other published examples of crystal structures of complexes of the two above mentioned ligands both coordinated to a transition metal.

The title complex is shown in Fig. 1. The Zn^II^ ion is in a slightly distorted trigonal-bipyramidal coordination geometry. In the crystal structure, there are weak hydrogen bonds formed between complex cations and perchlorate anions, and between symmetry related complex cations (Fig. 2). In addition there also exists interactions between Cl—O bonds and π-rings systems and between C—N bonds and π-ring systems, and the relevant distances (Å) are as follows: Cl1—O2···*Cg*1^i^ = 3.236 (3), Cl1—O2···*Cg*1^i^_perp_ = 3.120; Cl1—O4···*Cg*2^i^ = 3.179 (3), Cl1—O4···*Cg*2^i^_perp_ = 3.111; C20—N6···*Cg*2^ii^ = 3.381 (4), C20—N6···*Cg*2^ii^_perp_ = 3.360; C20—N6···*Cg*1^iii^ = 3.453 (4), C20—N6···*Cg*1^iii^_perp_ = 3.156; C20—N6···*Cg*3^iii^ = 3.761 (3), C20—N6···*Cg*3^iii^_perp_ = 3.365; (*Cg*1, *Cg*2 and *Cg*3 are the centroids of N2/C1—C5 ring, N1/C8—C12 ring and N3/C14—C18 ring, respectively; A—B···Cgj_perp_ is the perpendicular distance from B atom to ring j; symmetry codes: (i) *x*,-1 + *y*,*z*; (ii) 1 - *x*,2 - *y*,-*z*; (iii) 1 - *x*,1/2 + *y*,1/2 - *z*).

### Experimental

A 10 ml me thanol solution of tris[(2-pyridyl)methylene]amine (0.2103 g, 0.72 mmol) was added into 20 ml H_2_O solution containing Zn(ClO_4_)6H_2_O (0.2621 g, 0.70 mmol) and sodium dicyanamide (0.0645 g, 0.72 mmol), and the mixture was stirred for a few minutes. Colorless single crystals were obtained after the filtrate had been allowed to stand at room temperature for 10 d.

### Refinement

The central N atom of the dicyanamide ligand is disorded over two sites in a 0.40 (7): 60 (7) ratio (sum constrained to unity). H atoms were placed in calculated positions (C—H = 0.97 Å for methylene group and C—H = 0.93 Å for other H atoms) and refined as riding with *U*_iso_ = 1.2 *U*_eq_(C).

### Crystal data

**Table:**

[Zn(C_2_N_3_)(C_18_H_18_N_4_)]ClO_4_	*F*_000_ = 1064
*M**_r_* = 521.23	*D*_x_ = 1.563 Mg m^−^^3^
Monoclinic, *P*2_1_/*c*	Mo *K*α radiation λ = 0.71073 Å
Hall symbol: -P 2ybc	Cell parameters from 2616 reflections
*a* = 13.931 (2) Å	θ = 2.3–22.0º
*b* = 10.8578 (18) Å	µ = 1.27 mm^−^^1^
*c* = 14.653 (2) Å	*T* = 298 (2) K
β = 91.590 (3)º	Block, colorless
*V* = 2215.5 (6) Å^3^	0.30 × 0.20 × 0.18 mm
*Z* = 4	

### Data collection

**Table:**

Bruker SMART APEX CCD diffractometer	4800 independent reflections
Radiation source: fine-focus sealed tube	3352 reflections with *I* > 2σ(*I*)
Monochromator: graphite	*R*_int_ = 0.035
*T* = 298(2) K	θ_max_ = 27.0º
φ and ω scans	θ_min_ = 2.3º
Absorption correction: multi-scan(SADABS; Sheldrick, 1996)	*h* = −15→17
*T*_min_ = 0.702, *T*_max_ = 0.803	*k* = −13→12
12346 measured reflections	*l* = −18→16

### Refinement

**Table:**

Refinement on *F*^2^	Secondary atom site location: difference Fourier map
Least-squares matrix: full	Hydrogen site location: inferred from neighbouring sites
*R*[*F*^2^ > 2σ(*F*^2^)] = 0.043	H-atom parameters constrained
*wR*(*F*^2^) = 0.106	*w* = 1/[σ^2^(*F*_o_^2^) + (0.053*P*)^2^] where *P* = (*F*_o_^2^ + 2*F*_c_^2^)/3
*S* = 0.99	(Δ/σ)_max_ = 0.001
4800 reflections	Δρ_max_ = 0.36 e Å^−^^3^
308 parameters	Δρ_min_ = −0.25 e Å^−^^3^
1 restraint	Extinction correction: none
Primary atom site location: structure-invariant direct methods	

### Special details

**Table:**

Geometry. All e.s.d.'s (except the e.s.d. in the dihedral angle between two l.s. planes) are estimated using the full covariance matrix. The cell e.s.d.'s are taken into account individually in the estimation of e.s.d.'s in distances, angles and torsion angles; correlations between e.s.d.'s in cell parameters are only used when they are defined by crystal symmetry. An approximate (isotropic) treatment of cell e.s.d.'s is used for estimating e.s.d.'s involving l.s. planes.
Refinement. Refinement of *F*^2^ against ALL reflections. The weighted *R*-factor *wR* and goodness of fit *S* are based on *F*^2^, conventional *R*-factors *R* are based on *F*, with *F* set to zero for negative *F*^2^. The threshold expression of *F*^2^ > σ(*F*^2^) is used only for calculating *R*-factors(gt) *etc*. and is not relevant to the choice of reflections for refinement. *R*-factors based on *F*^2^ are statistically about twice as large as those based on *F*, and *R*- factors based on ALL data will be even larger.

### Fractional atomic coordinates and isotropic or equivalent isotropic displacement parameters (Å^2^)

**Table:**

	*x*	*y*	*z*	*U*_iso_*/*U*_eq_	Occ. (<1)
C1	0.0860 (2)	0.9139 (3)	0.2853 (2)	0.0612 (9)	
H1	0.0337	0.8627	0.2947	0.073*	
C2	0.1044 (3)	1.0115 (4)	0.3423 (2)	0.0697 (10)	
H2	0.0648	1.0272	0.3910	0.084*	
C3	0.1813 (3)	1.0854 (3)	0.3270 (2)	0.0674 (9)	
H3	0.1952	1.1517	0.3654	0.081*	
C4	0.2375 (2)	1.0607 (3)	0.2547 (2)	0.0545 (8)	
H4	0.2894	1.1122	0.2439	0.065*	
C5	0.14649 (19)	0.8927 (3)	0.21361 (19)	0.0461 (7)	
C6	0.1355 (2)	0.7823 (3)	0.1528 (2)	0.0562 (8)	
H6A	0.1669	0.7122	0.1816	0.067*	
H6B	0.0678	0.7629	0.1445	0.067*	
C7	0.1151 (2)	0.8761 (3)	0.0017 (2)	0.0629 (9)	
H7A	0.0784	0.9345	0.0367	0.076*	
H7B	0.0702	0.8217	−0.0301	0.076*	
C8	0.1729 (2)	0.9441 (3)	−0.0664 (2)	0.0519 (7)	
C9	0.1372 (3)	0.9713 (3)	−0.1530 (2)	0.0691 (9)	
H9	0.0769	0.9431	−0.1722	0.083*	
C10	0.1913 (4)	1.0397 (4)	−0.2098 (2)	0.0812 (11)	
H10	0.1684	1.0590	−0.2683	0.097*	
C11	0.2798 (3)	1.0797 (3)	−0.1801 (2)	0.0786 (11)	
H11	0.3177	1.1267	−0.2180	0.094*	
C12	0.3118 (3)	1.0497 (3)	−0.0937 (2)	0.0617 (8)	
H12	0.3719	1.0774	−0.0736	0.074*	
C13	0.2121 (2)	0.6901 (3)	0.0215 (2)	0.0628 (9)	
H13A	0.2159	0.7016	−0.0439	0.075*	
H13B	0.1676	0.6233	0.0324	0.075*	
C14	0.3093 (2)	0.6574 (3)	0.0606 (2)	0.0554 (8)	
C15	0.3413 (4)	0.5360 (3)	0.0654 (3)	0.0844 (12)	
H15	0.3010	0.4718	0.0469	0.101*	
C16	0.4314 (4)	0.5126 (4)	0.0972 (3)	0.1041 (15)	
H16	0.4535	0.4318	0.1008	0.125*	
C17	0.4900 (3)	0.6072 (4)	0.1240 (3)	0.0947 (14)	
H17	0.5526	0.5922	0.1448	0.114*	
C18	0.4545 (2)	0.7252 (3)	0.1195 (2)	0.0681 (9)	
H18	0.4936	0.7899	0.1392	0.082*	
C19	0.4657 (2)	1.1134 (3)	0.1232 (2)	0.0594 (8)	
C20	0.5964 (3)	1.2341 (3)	0.1587 (2)	0.0670 (9)	
Cl1	0.13251 (5)	0.26686 (7)	0.06625 (5)	0.0551 (2)	
N1	0.25963 (17)	0.9822 (2)	−0.03743 (15)	0.0474 (6)	
N2	0.22133 (15)	0.9657 (2)	0.19860 (14)	0.0425 (5)	
N3	0.36571 (17)	0.7501 (2)	0.08790 (16)	0.0500 (6)	
N4	0.17686 (15)	0.8038 (2)	0.06381 (15)	0.0461 (6)	
N5	0.42095 (18)	1.0295 (3)	0.11270 (19)	0.0626 (7)	
N6	0.6718 (3)	1.2592 (3)	0.1805 (2)	0.1036 (12)	
N7	0.5015 (14)	1.214 (2)	0.157 (3)	0.066 (7)	0.40 (7)
N7'	0.5148 (16)	1.2179 (15)	0.116 (3)	0.100 (5)	0.60 (7)
O1	0.1054 (2)	0.3925 (2)	0.06831 (17)	0.0854 (8)	
O2	0.08216 (17)	0.2017 (2)	0.13439 (16)	0.0821 (7)	
O3	0.23268 (17)	0.2569 (3)	0.0827 (2)	0.0980 (8)	
O4	0.1080 (2)	0.2177 (2)	−0.02084 (16)	0.0905 (8)	
Zn1	0.30405 (2)	0.92245 (3)	0.08979 (2)	0.04138 (12)	

### Atomic displacement parameters (Å^2^)

**Table:**

	*U*^11^	*U*^22^	*U*^33^	*U*^12^	*U*^13^	*U*^23^
C1	0.0467 (17)	0.086 (3)	0.0509 (19)	0.0100 (16)	0.0088 (14)	0.0197 (18)
C2	0.081 (3)	0.086 (3)	0.0423 (19)	0.024 (2)	0.0134 (17)	0.0048 (18)
C3	0.089 (3)	0.067 (2)	0.0456 (19)	0.0119 (19)	0.0039 (18)	−0.0042 (16)
C4	0.0615 (19)	0.056 (2)	0.0459 (18)	0.0023 (14)	−0.0017 (15)	0.0004 (14)
C5	0.0401 (15)	0.0575 (18)	0.0404 (16)	0.0022 (13)	−0.0029 (12)	0.0081 (13)
C6	0.0447 (17)	0.068 (2)	0.0564 (19)	−0.0167 (15)	0.0066 (14)	0.0053 (15)
C7	0.0424 (17)	0.090 (2)	0.056 (2)	−0.0105 (16)	−0.0095 (15)	0.0053 (18)
C8	0.0528 (18)	0.0588 (19)	0.0437 (17)	0.0085 (14)	−0.0041 (14)	0.0003 (14)
C9	0.083 (2)	0.076 (2)	0.048 (2)	0.0142 (19)	−0.0125 (18)	−0.0030 (18)
C10	0.123 (4)	0.075 (3)	0.045 (2)	0.025 (2)	−0.006 (2)	0.0083 (18)
C11	0.120 (3)	0.062 (2)	0.055 (2)	0.005 (2)	0.020 (2)	0.0158 (17)
C12	0.074 (2)	0.0515 (19)	0.061 (2)	−0.0001 (16)	0.0158 (17)	0.0026 (15)
C13	0.074 (2)	0.056 (2)	0.059 (2)	−0.0263 (17)	0.0047 (17)	−0.0095 (15)
C14	0.075 (2)	0.0442 (17)	0.0481 (17)	−0.0047 (15)	0.0153 (15)	−0.0035 (14)
C15	0.119 (4)	0.046 (2)	0.089 (3)	0.001 (2)	0.030 (3)	0.0011 (19)
C16	0.138 (4)	0.067 (3)	0.108 (4)	0.043 (3)	0.033 (3)	0.018 (3)
C17	0.090 (3)	0.100 (4)	0.095 (3)	0.048 (3)	0.007 (2)	0.012 (3)
C18	0.061 (2)	0.073 (2)	0.070 (2)	0.0125 (17)	0.0050 (18)	0.0036 (18)
C19	0.0489 (18)	0.0545 (18)	0.074 (2)	−0.0062 (12)	−0.0102 (16)	0.0012 (17)
C20	0.071 (2)	0.058 (2)	0.072 (2)	−0.0248 (18)	−0.0010 (19)	−0.0053 (17)
Cl1	0.0535 (4)	0.0568 (5)	0.0552 (5)	−0.0035 (3)	0.0042 (4)	0.0010 (4)
N1	0.0524 (14)	0.0476 (14)	0.0424 (13)	0.0022 (11)	0.0063 (11)	0.0037 (11)
N2	0.0419 (13)	0.0463 (13)	0.0391 (13)	0.0032 (10)	−0.0033 (10)	0.0028 (10)
N3	0.0524 (15)	0.0494 (14)	0.0484 (15)	0.0025 (11)	0.0044 (11)	0.0023 (11)
N4	0.0411 (13)	0.0553 (15)	0.0421 (13)	−0.0109 (11)	0.0025 (10)	0.0002 (11)
N5	0.0485 (15)	0.0609 (16)	0.0784 (19)	−0.0141 (11)	0.0000 (13)	−0.0056 (14)
N6	0.090 (2)	0.118 (3)	0.103 (3)	−0.050 (2)	0.010 (2)	−0.037 (2)
N7	0.060 (6)	0.040 (8)	0.096 (15)	−0.005 (5)	−0.014 (8)	0.007 (6)
N7'	0.089 (7)	0.082 (6)	0.128 (15)	−0.040 (5)	−0.042 (8)	0.052 (7)
O1	0.107 (2)	0.0644 (16)	0.0849 (19)	0.0212 (14)	0.0130 (15)	0.0035 (13)
O2	0.0737 (16)	0.1009 (19)	0.0720 (16)	−0.0049 (13)	0.0058 (13)	0.0369 (14)
O3	0.0550 (15)	0.0947 (19)	0.144 (2)	−0.0051 (13)	0.0035 (15)	0.0138 (17)
O4	0.110 (2)	0.098 (2)	0.0639 (16)	−0.0235 (16)	0.0170 (14)	−0.0221 (14)
Zn1	0.03599 (18)	0.0427 (2)	0.0454 (2)	−0.00542 (13)	0.00088 (13)	−0.00063 (14)

### Geometric parameters (Å, °)

**Table:**

C1—C2	1.368 (5)	C13—C14	1.498 (4)
C1—C5	1.384 (4)	C13—H13A	0.9700
C1—H1	0.9300	C13—H13B	0.9700
C2—C3	1.362 (5)	C14—N3	1.332 (4)
C2—H2	0.9300	C14—C15	1.392 (5)
C3—C4	1.362 (4)	C15—C16	1.352 (6)
C3—H3	0.9300	C15—H15	0.9300
C4—N2	1.334 (3)	C16—C17	1.363 (6)
C4—H4	0.9300	C16—H16	0.9300
C5—N2	1.333 (3)	C17—C18	1.374 (5)
C5—C6	1.499 (4)	C17—H17	0.9300
C6—N4	1.458 (3)	C18—N3	1.336 (4)
C6—H6A	0.9700	C18—H18	0.9300
C6—H6B	0.9700	C19—N5	1.112 (4)
C7—N4	1.463 (4)	C19—N7	1.294 (17)
C7—C8	1.495 (4)	C19—N7'	1.331 (14)
C7—H7A	0.9700	C20—N6	1.122 (4)
C7—H7B	0.9700	C20—N7'	1.298 (16)
C8—N1	1.335 (4)	C20—N7	1.34 (2)
C8—C9	1.382 (4)	Cl1—O3	1.414 (2)
C9—C10	1.359 (5)	Cl1—O4	1.416 (2)
C9—H9	0.9300	Cl1—O1	1.416 (2)
C10—C11	1.367 (6)	Cl1—O2	1.424 (2)
C10—H10	0.9300	N1—Zn1	2.053 (2)
C11—C12	1.369 (5)	N2—Zn1	2.048 (2)
C11—H11	0.9300	N3—Zn1	2.059 (2)
C12—N1	1.334 (4)	N4—Zn1	2.215 (2)
C12—H12	0.9300	N5—Zn1	2.021 (3)
C13—N4	1.472 (4)		
			
C2—C1—C5	119.0 (3)	C16—C15—C14	119.3 (4)
C2—C1—H1	120.5	C16—C15—H15	120.4
C5—C1—H1	120.5	C14—C15—H15	120.4
C3—C2—C1	119.4 (3)	C15—C16—C17	120.0 (4)
C3—C2—H2	120.3	C15—C16—H16	120.0
C1—C2—H2	120.3	C17—C16—H16	120.0
C2—C3—C4	118.9 (3)	C16—C17—C18	118.5 (4)
C2—C3—H3	120.5	C16—C17—H17	120.7
C4—C3—H3	120.5	C18—C17—H17	120.7
N2—C4—C3	122.7 (3)	N3—C18—C17	122.3 (4)
N2—C4—H4	118.6	N3—C18—H18	118.9
C3—C4—H4	118.6	C17—C18—H18	118.9
N2—C5—C1	121.4 (3)	N5—C19—N7	162.3 (19)
N2—C5—C6	116.4 (2)	N5—C19—N7'	166.9 (17)
C1—C5—C6	122.0 (3)	N6—C20—N7'	166.3 (18)
N4—C6—C5	111.5 (2)	N6—C20—N7	163.6 (17)
N4—C6—H6A	109.3	O3—Cl1—O4	109.71 (18)
C5—C6—H6A	109.3	O3—Cl1—O1	109.44 (17)
N4—C6—H6B	109.3	O4—Cl1—O1	108.92 (16)
C5—C6—H6B	109.3	O3—Cl1—O2	110.20 (16)
H6A—C6—H6B	108.0	O4—Cl1—O2	109.48 (16)
N4—C7—C8	111.2 (2)	O1—Cl1—O2	109.07 (16)
N4—C7—H7A	109.4	C12—N1—C8	118.5 (3)
C8—C7—H7A	109.4	C12—N1—Zn1	125.4 (2)
N4—C7—H7B	109.4	C8—N1—Zn1	115.97 (19)
C8—C7—H7B	109.4	C5—N2—C4	118.6 (3)
H7A—C7—H7B	108.0	C5—N2—Zn1	116.86 (19)
N1—C8—C9	121.7 (3)	C4—N2—Zn1	124.6 (2)
N1—C8—C7	116.1 (2)	C14—N3—C18	118.9 (3)
C9—C8—C7	122.1 (3)	C14—N3—Zn1	116.6 (2)
C10—C9—C8	119.2 (4)	C18—N3—Zn1	124.2 (2)
C10—C9—H9	120.4	C6—N4—C7	113.7 (2)
C8—C9—H9	120.4	C6—N4—C13	112.7 (2)
C9—C10—C11	119.3 (3)	C7—N4—C13	112.7 (2)
C9—C10—H10	120.4	C6—N4—Zn1	105.89 (16)
C11—C10—H10	120.4	C7—N4—Zn1	104.41 (17)
C10—C11—C12	119.1 (3)	C13—N4—Zn1	106.58 (17)
C10—C11—H11	120.4	C19—N5—Zn1	160.0 (3)
C12—C11—H11	120.4	C19—N7—C20	120.9 (14)
N1—C12—C11	122.2 (3)	C20—N7'—C19	121.4 (13)
N1—C12—H12	118.9	N5—Zn1—N2	101.94 (10)
C11—C12—H12	118.9	N5—Zn1—N1	100.96 (11)
N4—C13—C14	110.3 (2)	N2—Zn1—N1	118.19 (9)
N4—C13—H13A	109.6	N5—Zn1—N3	100.98 (10)
C14—C13—H13A	109.6	N2—Zn1—N3	117.58 (9)
N4—C13—H13B	109.6	N1—Zn1—N3	112.99 (9)
C14—C13—H13B	109.6	N5—Zn1—N4	179.41 (10)
H13A—C13—H13B	108.1	N2—Zn1—N4	78.60 (9)
N3—C14—C15	121.0 (3)	N1—Zn1—N4	78.95 (9)
N3—C14—C13	117.1 (3)	N3—Zn1—N4	78.55 (9)
C15—C14—C13	121.9 (3)		
			
C5—C1—C2—C3	0.2 (5)	C14—C13—N4—Zn1	34.9 (3)
C1—C2—C3—C4	0.6 (5)	N7—C19—N5—Zn1	63 (5)
C2—C3—C4—N2	−1.0 (5)	N7'—C19—N5—Zn1	−71 (7)
C2—C1—C5—N2	−0.6 (4)	N5—C19—N7—C20	138.7 (19)
C2—C1—C5—C6	175.2 (3)	N7'—C19—N7—C20	−61 (3)
N2—C5—C6—N4	−27.9 (3)	N6—C20—N7—C19	−134 (2)
C1—C5—C6—N4	156.1 (3)	N7'—C20—N7—C19	65 (3)
N4—C7—C8—N1	−33.0 (4)	N6—C20—N7'—C19	141 (3)
N4—C7—C8—C9	150.0 (3)	N7—C20—N7'—C19	−62 (2)
N1—C8—C9—C10	−0.7 (5)	N5—C19—N7'—C20	−142 (3)
C7—C8—C9—C10	176.1 (3)	N7—C19—N7'—C20	66 (3)
C8—C9—C10—C11	0.1 (6)	C19—N5—Zn1—N2	−60.5 (8)
C9—C10—C11—C12	0.1 (6)	C19—N5—Zn1—N1	61.7 (8)
C10—C11—C12—N1	0.2 (5)	C19—N5—Zn1—N3	178.0 (8)
N4—C13—C14—N3	−30.8 (4)	C5—N2—Zn1—N5	−167.30 (19)
N4—C13—C14—C15	151.5 (3)	C4—N2—Zn1—N5	12.5 (2)
N3—C14—C15—C16	−0.8 (5)	C5—N2—Zn1—N1	83.2 (2)
C13—C14—C15—C16	176.8 (4)	C4—N2—Zn1—N1	−97.0 (2)
C14—C15—C16—C17	−0.1 (7)	C5—N2—Zn1—N3	−58.0 (2)
C15—C16—C17—C18	1.3 (7)	C4—N2—Zn1—N3	121.8 (2)
C16—C17—C18—N3	−1.6 (6)	C5—N2—Zn1—N4	12.47 (18)
C11—C12—N1—C8	−0.8 (5)	C4—N2—Zn1—N4	−167.7 (2)
C11—C12—N1—Zn1	175.7 (2)	C12—N1—Zn1—N5	14.7 (3)
C9—C8—N1—C12	1.0 (4)	C8—N1—Zn1—N5	−168.7 (2)
C7—C8—N1—C12	−176.0 (3)	C12—N1—Zn1—N2	124.8 (2)
C9—C8—N1—Zn1	−175.8 (2)	C8—N1—Zn1—N2	−58.7 (2)
C7—C8—N1—Zn1	7.2 (3)	C12—N1—Zn1—N3	−92.3 (2)
C1—C5—N2—C4	0.2 (4)	C8—N1—Zn1—N3	84.2 (2)
C6—C5—N2—C4	−175.8 (2)	C12—N1—Zn1—N4	−164.7 (3)
C1—C5—N2—Zn1	−180.0 (2)	C8—N1—Zn1—N4	11.9 (2)
C6—C5—N2—Zn1	4.0 (3)	C14—N3—Zn1—N5	−170.4 (2)
C3—C4—N2—C5	0.6 (4)	C18—N3—Zn1—N5	15.8 (3)
C3—C4—N2—Zn1	−179.2 (2)	C14—N3—Zn1—N2	79.8 (2)
C15—C14—N3—C18	0.6 (4)	C18—N3—Zn1—N2	−94.1 (3)
C13—C14—N3—C18	−177.2 (3)	C14—N3—Zn1—N1	−63.4 (2)
C15—C14—N3—Zn1	−173.6 (2)	C18—N3—Zn1—N1	122.8 (2)
C13—C14—N3—Zn1	8.6 (3)	C14—N3—Zn1—N4	9.3 (2)
C17—C18—N3—C14	0.7 (5)	C18—N3—Zn1—N4	−164.6 (3)
C17—C18—N3—Zn1	174.4 (3)	C6—N4—Zn1—N2	−25.66 (18)
C5—C6—N4—C7	−79.4 (3)	C7—N4—Zn1—N2	94.61 (19)
C5—C6—N4—C13	150.8 (2)	C13—N4—Zn1—N2	−145.91 (19)
C5—C6—N4—Zn1	34.7 (3)	C6—N4—Zn1—N1	−147.68 (19)
C8—C7—N4—C6	153.5 (3)	C7—N4—Zn1—N1	−27.42 (18)
C8—C7—N4—C13	−76.7 (3)	C13—N4—Zn1—N1	92.06 (19)
C8—C7—N4—Zn1	38.6 (3)	C6—N4—Zn1—N3	95.86 (18)
C14—C13—N4—C6	−80.8 (3)	C7—N4—Zn1—N3	−143.9 (2)
C14—C13—N4—C7	148.9 (2)	C13—N4—Zn1—N3	−24.39 (18)

### Hydrogen-bond geometry (Å, °)

**Table:**

*D*—H···*A*	*D*—H	H···*A*	*D*···*A*	*D*—H···*A*
C3—H3···O4^i^	0.93	2.53	3.272 (4)	138
C7—H7B···O2^ii^	0.97	2.59	3.455 (4)	148
C10—H10···O1^iii^	0.93	2.58	3.511 (5)	179
C13—H13A···N6^iv^	0.97	2.61	3.457 (5)	146
C16—H16···N7'^v^	0.93	2.48	3.412 (18)	178

Symmetry codes: (i) *x*, −*y*+3/2, *z*+1/2; (ii) −*x*, −*y*+1, −*z*; (iii) *x*, −*y*+3/2, *z*−1/2; (iv) −*x*+1, −*y*+2, −*z*; (v) *x*, *y*−1, *z*.
